# Technology Enhanced Assessment (TEA) in COVID 19 Pandemic

**DOI:** 10.12669/pjms.36.COVID19-S4.2795

**Published:** 2020-05

**Authors:** Rehan Ahmed Khan, Masood Jawaid

**Affiliations:** 1 Prof. Rehan Ahmed Khan MBBS(Pak), FCPS (Pak), FRCS (Ire), JM-HPE (Ned), MSc HPE (UK) Professor of Surgery, Assistant Dean Medical Education, Riphah International University, Islamabad, Pakistani; 2 Dr. Masood Jawaid MBBS, MCPS, MRCS (Glasg), FCPS (Surg), MHPE. Director Medical Affairs and Pharmacy Services, PharmEvo, Consultant Surgeon, DSH Karachi, Pakistani

**Keywords:** Online assessment, COVID 19, Technology enhanced assessment, Pandemic

## Abstract

Online teaching and learning is not a new phenomenon. For the last many years, it has been mainly used as a part of face to face teaching. Assessment is an essential part of teaching and learning, as it establishes the achievement of course learning outcomes by the students. Computer-based assessment is in place for a long time now, however, online assessments have been less practiced. This is because of the issues of validity, reliability and dishonesty. During the COVID 19 pandemic, the educational environment has taken a paradigm shift in many medical schools, both nationally and internationally. This situation demands a method of assessment that is safe, valid, reliable, acceptable, feasible and fair. This paper describes the different formats of online assessment and their application in formative and summative assessments during and after the COVID 19 pandemic.

## BACKGROUND

Assessment is the measurement of learning of a student. It can be divided into ‘assessment of learning’ (summative) and ‘assessment for learning (formative). Summative assessment is used for pass/fail decisions, whereas formative assessment for providing feedback.^1^ Cees Van der Vleuten describes programmatic assessment as low, mid and high stakes, collectively called as Programmatic assessment.^2^ Each type has both formative and summative components and is assigned a certain weightage. In Pakistan, majority of the medical schools use formative or continuous assessment (10-20% weightage) and summative or end of year assessments called Professionals (80-90% weightage). However, in some schools, where programmatic assessment is a method of assessment, low stakes assessment is 10-20%, mid stake 30-40% and high stake 50-60%.^3^ Whatever the method used, the assessment of student comprises of measuring knowledge, skill and attitude. The knowledge is usually assessed through multiple choice questions, short essay questions and long essay type questions. Skill is tested through OPSE, OSCE, Practical, Vivas, Short and Long cases.^4^ Pre COVID 19, these domains of learning were assessed face to face (f2f).^5^ With the arrival of COVID 19 pandemic, there has been a paradigm shift from traditional f2f teaching and learning to online technology enhanced learning.

As predicted this transformation in the educational environment will bring long-lasting effects on teaching and learning, assessment procedures and methods also require a change. This commentary is aimed at describing different assessment options that can be used online, taking into account the educational environment in a pandemic situation.

### Online Assessment Platforms

Online Teaching and Learning is delivered either asynchronously or synchronously, where the latter is done in real-time, face to face and online.

### Asynchronous methods of assessment

In asynchronous method of assessment, which is not done in realtime, assignments and portfolios can be used to assess knowledge and skills.

### Assignments.

Assignments can assess higher order thinking which includes critical thinking and problem-solving ability of the student. It is however important that a uniform rubric be developed to check assignments and detailed qualitative feedback is given to the students to improve their learning.^6^

### Assessment Portfolios

A portfolio is an evidence of a task performed by the student along with reflection on it. It can be used to assess skills of the students through online submission of recorded videos of the tasks performed by them. These tasks can be about history and examination of patients, counselling, breaking bad news etc., on which the teachers can provide feedback.^7^

### Synchronous methods of assessment

Online, real-time assessments can be used to reproduce the traditional methods of assessment.^6^

### Multiple Choice Questions.

To assess knowledge, multiple choice questions (single best, one correct, extended matching etc.) can be given online to the students on a predetermined date and for fixed duration. They can assess low to high order cognitive thinking skills depending upon their construct.

### Open Book Exams

The aim of this method of assessment is to assess the ability of students to analyze and solve a problem, assess critical thinking and creativity. With open book exams taken in real time, the issues of cheating can be minimized.^8^

### Objectively Structured Practical/Clinical Examination

Similarly, OSPE or OSCE’s can be taken online using the ‘essay’ format in different Learning management systems (LMS). LMS provides the examiner to insert a picture or a video, on which questions can be asked from the candidate. Spotting in anatomy can be done through image hot spots. Items can be embedded in videos on clinical methods or procedures to assess the clinical competency of the student.

### Vivas (Online)

Clinical competence is most difficult to assess online and specifically in the time of Covid19 pandemic because of social distancing and stay at home restrictions. However, vivas can be conducted using online video calls to assess decision making, critical thinking and clinical reasoning skills of a student based on a clinical case. Teacher can also simulate as a patient, from whom a student can take history and inquire about relevant examination.

### Types of online platforms

There are several online platforms available for assessment having unique advantages and limitations.^7^ For example, large scale student assessment of MCQs can be done through Google Forms.^9^ It can be timed, and students can get real time feedback on their responses. Institutions using LMS such as MOODLE can use advanced assessment settings for different question types, such as shuffling the items and their options, using sequential or free navigation etc.^10^ To prevent cheating in real-time exams, assessment software companies also offer technology based invigilation. Though such advance features make online assessment more reliable in terms of preventing cheating, the cost is much higher.^11^

## CONCLUSION

Can we shy away from online teaching, learning and assessment anymore? we think the answer is ‘No’. The educationally advanced countries had embraced these techniques earlier. So they faced less difficulty in imparting education online in COVID 19 pandemic as compared to us where lack of resources, infrastructure, training and acceptability had hindered this form of education for a long time. It’s time to move in the right direction by adapting technology enhanced learning and assessment for our educational system.
